# 

**Published:** 1897-09-18

**Authors:** 


### EARLY EDITION.

**Notices of Births, Deaths, and Marriages** are inserted in this column at a charge of 2s. 6d. for an announcement not exceeding four lines.

### NOTICE TO CORRESPONDENTS.

All MS., Letters, Books for Review, and other matters intended for the Editor should be addressed The Editor, "The Hospital," The Hospital Building, 28 & 29, Southampton Street, Strand, W.C.

The Editor cannot undertake to return rejected MS., even when accompanied by stamped directed envelope.

**Notice to Advertisers and Subscribers.**

All Advertisements, Orders for Copies of The Hospital, and Business Communications should be addressed to **THE MANAGER, (Not to The Editor),** The Hospital, The Hospital Building, 28 & 29, Southampton Street, Strand, London, W.C.

The Cost of Subscription, post free, is as follows:—Per week, 2½d.; per quarter, 2s. 8d.; per half-year, 5s. 3d.; per annum, 10s. 6d. Foreign:—Per annum, 15s. 2d.; payable, in all cases, in advance.

**NOTICE.—Vol. XXII. of "The Hospital" (April, 1897, to September, 1897), in handsome cloth binding, will be on sale at the Office of this Journal early in October, price 7s. 6d. Cases for binding the Numbers contained in this Volume, 1s. 6d. Index to Vol. XXII., 2½d., post free.**

**The Monthly Part for October will be ready on October 1. Price 6d., by post 8d.**

### BOOKS RECEIVED.

THE REDMAN PUBLISHING COMPANY.

"The Diseases of Women." By J. Bland Sutton, F.R.C.S. Eng Arthur E. Giles, M.D., B.Sc.Lond., F.R.C.S.Edin.

SAMPSON LOW, MARSTON, AND CO.

"Twentieth Century Practice of Medicine. Ed. by Thos. L. Stedman, M.D.

J. WRIGHT AND CO. (BRISTOL).

"Our Baby" (5th Ed.) By Mrs. Langton Hewer.

DIGBY, LONG, AND CO.

"The Worship of Lucifer." By Mina Sandeman.

"WESTERN MAIL" (LIMITED) (CARDIFF).

"Handbook to the Workman's Compensation Act, 1897. By M. Roberts-Jones.

THE SCIENTIFIC PRESS.

"Elementary Physiology for Nurses." Mr. C. F. Marshall, M.D., B.Sc., F.R.C.S.

"The Midwives' Pocket Book" (The Burdett Series, No. 3). By Honnor Morten.

CASSELL AND CO.

"The Queen's London." Part 1.

**Periodicals and Pamphlets.**—The Westminster Review, The Practitioner, The Humanitarian, Cassell's Family Magazine, Girls' Own Paper, Open Court, Literary Digest, London, Eureka, The Planter, Industries and Iron, Local Government Journal, The Melbourne Sun, The New Age, Knowledge, Electrical Review, The Vegetarian.

### The Student of Medicine.

**APPOINTMENTS.**

E. B. Bostock, M.B., B.S.Lond., M.R.C.S., L.R.C.P., appointed House Physician to the General Hospital, Birmingham. T. T. Cockill, M.R.C.S., L.R.C.P., appointed Assistant Honorary Physician to the North Staffordshire Infirmary. J. G. B. Coleman, M.R.C.S., L.R.C.P., appointed Medical Officer of the Sutton Bridge District of the Holbeach Union. W. Curling-Hayward, M.R.C.S., L.R.C.P., appointed House Physician to Charing Cross Hospital. W. Dixon, M.B., C.M., appointed Medical Officer of the No. 2 Hougham District of the Dover Union. W. C. Grosvenor, M.A., M.D., appointed Surgeon to the Royal Schools for the Deaf and Dumb, Old Trafford, Manchester. G. Hunter, M.D.Edin., appointed Medical Officer of the Aberdeen Dispensary. G. Lawrence, M B., C.M.Edin., appointed Resident Medical Assistant to the Dundee Infirmary. D. Lloyd, L.R.C.P.& S.Edin., appointed Medical Officer for the Newcastle Emlyn Rural District Council. A. M'Dougall, M.B., Ch.B.Vic., M.R.C.S., appointed Resident Medical Officer at the Manchester Royal Infirmary. E. J. Marmion, M.B., L.F.P.S. Glas., appointed Medical Officer of Health of the Milton Urban District Council. T. Morgan, L.R.C.P.I., L.R.C.S.I., appointed Medical Officer of Health to the Borough of Montgomery. W. H. Morgan, M.R.C.S., L.R.C.P., appointed House Surgeon to the Charing Cross Hospital. J. E. O'Connor, M.B., B.Ch., appointed Medical Officer of Health for the Borough of Lowestoft. E. C. Osborn, L.R.C.P., appointed First Assistant Medical Officer to the Kensington Infirmary. A. W. Pearse, M.R.C.S., L.R.C.P., appointed Second Assistant Medical Officer to the Kensington Infirmary. H. H. Phillips, L.R.C.P.Lond., M.R.C.S.Eng., appointed Divisional Surgeon to Police (P Division) stationed at Penge. H. H. Scott, M.R.C.S., L.R.C.P.Lond., appointed Resident House Surgeon to the Teignmouth Hospital, South Devon. H. T. Wickham, M D. Edin., re-appointed Medical Officer for the Second District of the Newport Pagnell Union. W. W. Woolliscroft, M.R.C.S., L R.C.P., appointed House Surgeon to Charing Cross Hospital. J. Maclaren, M.B., C.M.Edin., appointed Medica, Officer of the No. 3 District of the Wortley Union. F. C. Madden M.B., B.S.Melb., F.R.C.S.Eng., appointed Medical Superintendent to the Hospital for Sick Children, Great Ormond Street, W.C. A. L. Mathieson, M.B.Glasg.&C.M., appointed Medical Officer of the Children's Homes, Sheffield. C. Munden. M.R.C.S.Eng., L.S.A., appointed Medical Officer of the No. 2 District of the Chard Union. W. A. Murray, B.A.Camb., M.B., appointed Medical Officer of the Carlton and Drax District of the Selby Union. R. E. Newton, M.B., C.M.Glasg., appointed Medical Officer of the Fransham District of the Mitford and Launditch Union. J. J. Perkins, M.A., M.B.Cantab., M.R.C.P. Lond., appointed Assistant Physician to the City of London Hospital for Diseases of the Chest. W. H. Pimblett, M.B., C.M.Edin., appointed Medical Officer of the No. 7 District of the Preston Union. R. Pinhorn, L.R.C.P.Lond., M.R.C.S.Eng., L.S.A., appointed Honorary Medical Officer to the Dover Hospital. W. E. Thomson, M.A., M.D., appointed Examiner in Physiology to the University of St. Andrews. W. Simpson, M.B., C.M., appointed Assistant Medical Officer to the Newcastle-on-Tyne Union Workhouse. B. Sweeton, M.B., C.M. Edin., appointed Medical Officer of the Mexborough and Bolton-upon-Dearne Districts of the Doncaster Union. R H. Wilkin. M.R.C.S., L.R.C.P., appointed Medical Officer to the No. 3 District of the Newmarket Union. W. M. Woodhouse, M.R.C.S., L.R.C.P., appointed Medical Officer of the Vale District of the Wantage Union.

